# The Metalloporphyrin Antioxidant, MnTE-2-PyP, Inhibits Th2 Cell Immune Responses in an Asthma Model

**DOI:** 10.3390/ijms13089785

**Published:** 2012-08-06

**Authors:** Paiboon Jungsuwadee, Michael R. Weaver, Fabienne Gally, Rebecca E. Oberley-Deegan

**Affiliations:** 1 Department of Medicine, National Jewish Health, Denver, CO 80206, USA; E-Mails: weaverm@njhealth.org (M.R.W.); gallyf@njhealth.org (F.G.); oberleyr@njhealth.org (R.E.O.-D.); 2 Department of Biopharmaceutical Sciences, College of Pharmacy, Roosevelt University, Schaumburg Campus, Schaumburg, IL 60173, USA; 3 Department of Medicine, University of Colorado Health Science Center, Aurora, CO 80045, USA

**Keywords:** MnTE-2-PyP, OVA, dendritic cells, Th2 cells, asthma, inflammation

## Abstract

MnTE-2-PyP, a superoxide dismutase mimetic, inhibited OVA-induced airway inflammation in mice suggesting an effect on Th2 responsiveness. Thus, we hypothesized that MnTE-2-PyP may alter dendritic cell-Th2 interactions. Bone marrow derived dendritic cells (DC) and OVA_323–339_-specific Th2 cells were cultured separately in the presence or absence of MnTE-2-PyP for 3 days prior to the co-culturing of the two cell types in the presence of an OVA_323–339_ peptide and in some cases stimulated with CD3/CD28. MnTE-2-PyP-pretreated DC inhibited IL-4, IL-5 and IFNγ production and inhibited Th2 cell proliferation in the DC-Th2 co-culturing system in the presence of the OVA_323–339_ peptide. Similar results were obtained using the CD3/CD28 cell-activation system; the addition of MnTE-2-PyP inhibited Th2 cell proliferation. MnTE-2-PyP suppressed CD25 expression on OVA-specific Th2 cells, which implied that MnTE-2-PyP can inhibit the activation of Th2 cells. MnTE-2-PyP also down-regulated co-stimulatory molecules: CD40, CD80 and CD86 on immature DC. Our studies suggest that the major mechanism by which MnTE-2-PyP inhibits airway inflammation is by acting on the DC and suppressing Th2 cell proliferation and activation.

## 1. Introduction

Asthma is an inflammatory disorder characterized by airway inflammation, reversible airway obstruction, and airway hyperresponsiveness [1,2]. Oxidative stress has long been postulated to play a major role in the pathogenesis of airway inflammation [3]. For example, inflammatory cells retrieved from asthmatic patients generate more reactive oxygen species (ROS) than cells from non-asthmatic controls upon stimulation with phorbol myristate acetate [4,5]. Spontaneous release of superoxide anions (O_2_^−•^) by bronchial alveolar cells after an *in vivo* allergen provocation is increased in asthmatics [6]. In addition, asthmatic patients demonstrate depressed levels of endogenous antioxidant defense system such as superoxide dismutase (SOD) and glutathione [7].

Our laboratory has developed a SOD mimetic, MnTE-2-PyP [chemical name: Manganese (III) *Meso*-Tetrakis-(*N*-Methylpyridinium-2-yl], that scavenges a variety of ROS: superoxide, lipid peroxide, peroxynitrite and hydrogen peroxide [8–12]. Not only is MnTE-2-PyP an antioxidant, it is also an anti-inflammatory agent. MnTE-2-PyP inhibits inflammation and injury induced by radiation, bleomycin and ischemia/reperfusion [13–15]. We have demonstrated that MnTE-2-PyP inhibits airway inflammation and airway hyperresponsiveness in a mouse model of OVA-induced airway inflammation [1]. Recruitment of eosinophils and T lymphocytes into the lung was greatly reduced following intratracheal administration of MnTE-2-PyP during OVA challenges, suggesting that MnTE-2-PyP dampens the adaptive immune response [1].

ROS have been implicated as signaling molecules that influence the maturation of the adaptive immune cells, specifically T cells [16]. ROS appear to promote a pro-inflammatory response and cause proliferation of Th1 cells. When ROS are inhibited in T cells, a Th17 cytokine profile is achieved, further demonstrating the important role that redox environment has on the maturation of the adaptive immune system [16]. In a murine model of autoimmune-induced diabetes, antigen-presenting cells (APC) isolated from mice treated *in vivo* with MnTE-2-PyP showed a reduced ability to support T cell proliferation, suggesting an inhibitory role of MnTE-2-PyP on APC function [17]. Tse *et al.*, also reported MnTE-2-PyP reduces the activation of CD8^+^ and CD4^+^ T cells and causes the reduction of TNF-α and IFNγ levels in a diabetic mouse model [18].

The adaptive immune system also plays a critical role in allergic asthma. In allergen-induced airway inflammatory disease, the major antigen presenting cell is the dendritic cell (DC), which resides in the airway mucosa. Upon exposure to an antigen, airway DC acquire the antigen and rapidly mature into potent APC after cognate interactions with T memory cells in the mucosa of the conducting airways. DC then migrate to the draining lymph nodes [19]. The subsequent DC-T cell interactions result in the recruitment of inflammatory cells into the lung tissue, leading to a local inflammatory response. In allergic asthma, Th2 cells play a central role in disease pathogenesis. In previous studies, we have shown that MnTE-2-PyP inhibits airway inflammation in a mouse allergic asthma model [1]. We hypothesize that MnTE-2-PyP suppresses Th2 immune responses by inhibiting antigen presentation and DC-Th2 cell interactions, resulting in impairment of Th2 cell function. In this study, we investigated the effect of MnTE-2-PyP on DC-induced production of Th2 cell cytokines, Th2 cell proliferation and studied the effect of MnTE-2-PyP on DC maturation.

## 2. Results and Discussion

### 2.1. MnTE-2-PyP Reduces IL-4 and IL-5 Production in DC-T Cell Responses

**Figure 1:**
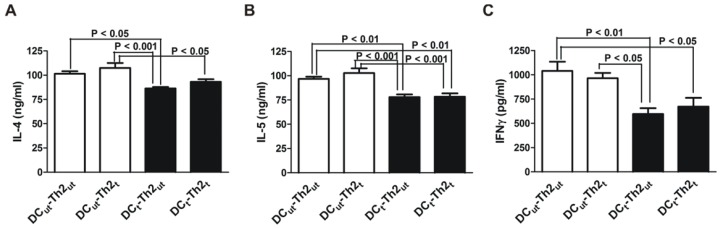
MnTE-2-PyP reduces cytokine production from co-cultured DC-Th2 cells. DC and Th2 cells were separately cultured in the absence (ut) or presence (t) of MnTE-2-PyP (30 μM). The DC and Th2 cells were then co-cultured in the presence of OVA_323–339_ peptide (1.5 μM) for 3 days. The supernatants were harvested and analyzed for (**A**) IL-4, (**B**) IL-5 and (**C**) IFNγ levels by ELISA. Data are represented as mean ± SEM from three independent experiments.

DC and OVA-specific Th2 cells were cultured in the presence or absence of MnTE-2-PyP for 3 days prior to co-culturing. To confirm that the OVA-specific Th2 cells display a Th2 phenotype we measured IL-4, IL-5 and IFNγ levels (Figure 1). As expected, under the MnTE-2-PyP naïve condition (DC_ut_-Th2_ut_), the cells produced large amounts of IL-4 and IL-5 and only small amounts of IFNγ. The ratio of Th2/Th1 cytokines was approximately 100-fold higher, confirming the Th2 phenotype of the CD4^+^ T cell (Figure 1, DC_ut_-Th2_ut_). We found that DC pretreated with MnTE-2-PyP inhibited both Th1 (IFNγ) and Th2 (IL-4 and IL-5) cytokine production by differentiated Th2 cells (Figure 1, closed bars). However, MnTE-2-PyP had no effect on cytokine production in Th2 cells (Figure 1, open bars).

**Figure 2:**
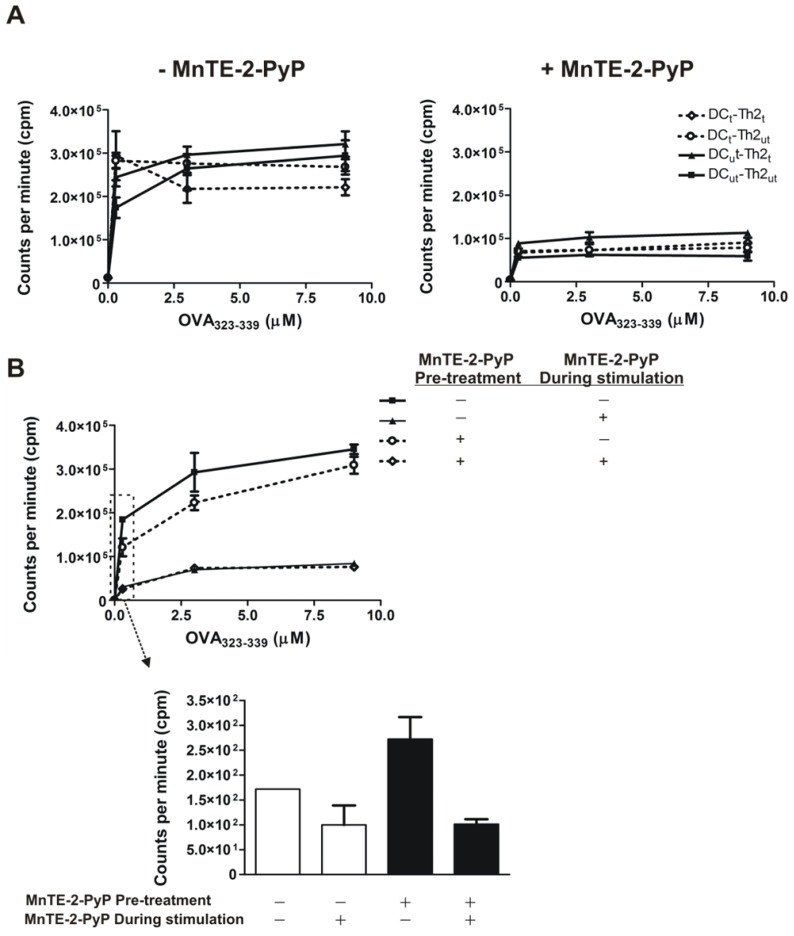
MnTE-2-PyP inhibits Th2 cell proliferation. DC and Th2 cells were co-cultured with OVA peptide for 3 days. [^3^H]thymidine was added during the last 18 h of culturing. Proliferation was measured by total uptake of [^3^H]thymidine (CPM). (**A**) MnTE-2-PyP treated- or untreated-DC and Th2 cells were co-cultured without (Left) and with MnTE-2-PyP (Right). (**B**) Th2 cells (pre-treated or untreated with MnTE-2-PyP) were cultured in anti-CD3 and anti-CD28 antibodies pre-coated plates with OVA_323–339_ peptide in the presence or absence of MnTE-2-PyP for 3 days. Th2 cell proliferation was measured by total uptake of [^3^H]thymidine. The bar graph inset shows the effect of MnTE-2-PyP on Th2 cell proliferation under CD3/CD28 stimulating system without OVA_323–339_ peptide. Data are represented as mean ± SEM from three independent experiments.

To examine the effect of MnTE-2-PyP on T cell proliferation, we stimulated Th2 cells in two different stimulating systems, one using DC and the other using an anti-CD3/CD28 antibodies pre-coated plate in the presence of various concentrations of OVA_323–339_ peptide for optimal stimulation. When we stimulated Th2 cells with DC, we found that MnTE-2-PyP pre-treatment of either DC or Th2 cells had no effect on the proliferation of Th2 cells (Figure 2A, Left). However, when MnTE-2-PyP was present during stimulation with DC and OVA_323–339_ peptide, a decrease in Th2 cell proliferation was observed (Figure 2A, Right). Similarly, in the anti-CD3/CD28 antibodies stimulating system, pre-treatment of Th2 cells with MnTE-2-PyP showed no inhibitory effect on cell proliferation. However, inclusion of MnTE-2-PyP during the period of Th2 stimulation resulted in the inhibition of Th2 cell proliferation (Figure 2B). Regardless of the treatment modalities, MnTE-2-PyP significantly inhibited cell proliferation (*p* < 0.01) independent of OVA_323–339_ peptide concentrations when the SOD mimetic was present in the culture media.

### 2.2. MnTE-2-PyP Down-Regulates CD25 on Th2 Cells

**Figure 3:**
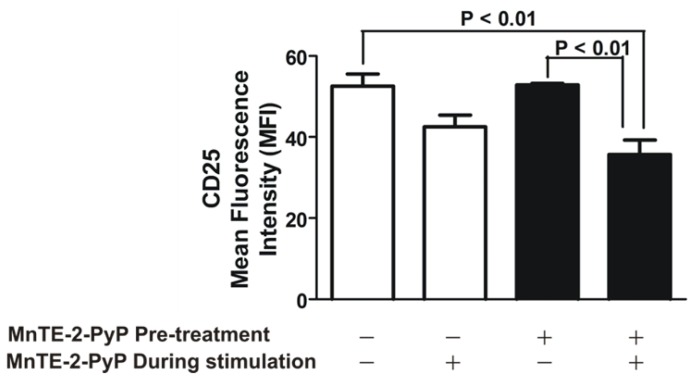
MnTE-2-PyP inhibits CD25 expression on Th2 cells. Th2 cells were either pre-treated with MnTE-2-PyP or left untreated, and then transferred to anti-CD3/CD28 antibodies pre-coated plate and OVA_323–339_ peptide (1.5 μM) for optimal stimulation in the presence or absence of MnTE-2-PyP (30 μM). Cells were labeled for CD25 expression and analyzed by FACS. Data are represented as mean ± SEM from three independent experiments.

We next determined the effect of MnTE-2-PyP on the activation of Th2 cells, by measuring the activated Th2 cell marker, CD25. We maintained OVA-specific Th2 cells in the presence or absence of MnTE-2-PyP for 3 days. We then transferred OVA-specific Th2 cells from each treatment group (MnTE-2-PyP *vs.* media alone) to an anti-CD3/CD28 antibodies pre-coated plate, and OVA_323–339_ peptide (1.5 μM) was added for optimal stimulation. The cells were maintained in the presence or absence of MnTE-2-PyP. The expression of CD25 was measured using FACS analysis. As shown in Figure 3, the expression of CD25 was significantly reduced by the SOD mimetic. Interestingly, in order to suppress the expression of CD25 on the Th2 cells, MnTE-2-PyP had to be present during stimulation. Pre-treatment of MnTE-2-PyP did not affect CD25 expression (Figure 3). Of note, the concentration of MnTE-2-PyP being used in our experiments did not affect cell survival as indicated by FACS analyses (data not shown).

### 2.3. Effect of MnTE-2-PyP on DC Surface Molecule Expression and Cytokine Production

**Figure 4:**
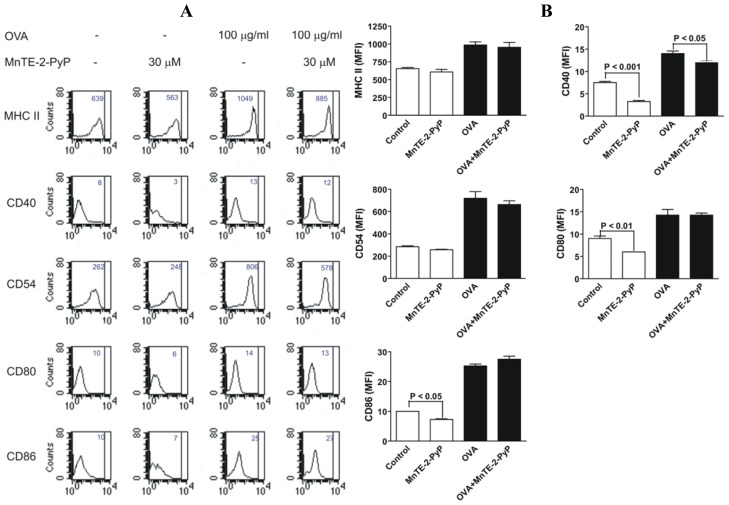
Co-stimulatory molecule expression on the surface of DC stimulated with OVA are reduced in the presence of MnTE-2-PyP. (**A**) Histograms of the FACS analyses of maturation markers on DC incubated with OVA for 42 h in the presence or absence of MnTE-2-PyP (30 μM). Mean fluorescence intensity (MFI) of the maturation marker is indicated at the upper right corner of each histogram. (**B**) Bar graphs of MFI of the DC maturation markers. Data are represented as MFI ± SEM from four independent experiments.

To investigate the inhibitory effect of MnTE-2-PyP on immature DC, bone marrow progenitor cells were cultured with GM-CSF and IL-4 to generate immature CD11c^+^ dendritic cells as described previously [20,21]. DC were either treated with MnTE-2-PyP or kept in the culture media without MnTE-2-PyP as a control. As shown in Figure 4A,B, we found that MnTE-2-PyP exerted no significant changes in the expressions of MHC class II molecules (Figure 4A,B). However, MnTE-2-PyP treated-DC significantly reduced the basal expressions of CD40, CD54, CD80 and CD86 (Figure 4A,B).

**Figure 5:**
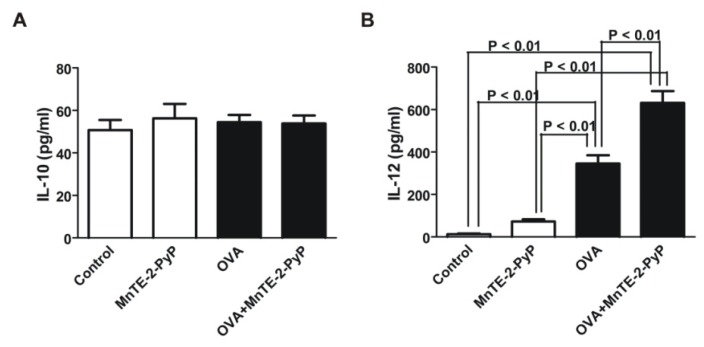
IL-12 p70 but not IL-10 production by OVA-stimulated DC is enhanced by MnTE-2-PyP. Bone marrow derived-DC were cultured in the presence or absence of OVA (100 μg/mL) for 42 h and with or without MnTE-2-PyP (30 μM). DC culture supernatants were harvested and assayed for (**A**) IL-10 and (**B**) IL-12 by ELISA. Data are represented as mean ± SEM from three independent experiments.

We also questioned whether MnTE-2-PyP could alter DC maturation. Because IL-12 expression has been identified as a specific marker of functionally activated DC [22], we then induced DC maturation by pulsing DC with endotoxin-depleted OVA and measured IL-10 and IL-12 cytokine production in the culture supernatants by ELISA. IL-10 expression levels remained the same regardless of treatment (Figure 5A). MnTE-2-PyP treatment enhanced basal level secretion of IL-12 by unstimulated DC (Figure 5B, Control *vs.* MnTE-2-PyP). The OVA-stimulated DC significantly increased IL-12 production (Figure 5B). Intriguingly, the concentrations of IL-12 in the media from MnTE-2-PyP-treated and OVA-stimulated DC were significantly higher than OVA-stimulated DC without the antioxidant treatment (Figure 5B, OVA *vs.* OVA-MnTE-2-PyP).

In this study, we demonstrate that the superoxide dismutase mimetic, MnTE-2-PyP, profoundly affects Th2 immune responses, not only by reducing the secretion of the Th1 cytokine IFNγ and Th2 cytokines IL-4 and IL-5 by T cells, but by preventing the up-regulation of co-stimulatory molecules e.g., CD40, CD80 and CD86 especially on immature DC. In addition, MnTE-2-PyP inhibits Th2 cell proliferation and inhibits the activated Th2 cell marker, CD25, on Th2 cells. The fact that MnTE-2-PyP inhibits both Th1 and Th2 cytokines suggests that MnTE-2-PyP inhibits cytokine production regardless of CD4^+^ T-helper cell phenotype. In allergic asthma models, IL-4 is known to be associated with airway hyperresponsiveness, and IL-5 is associated with eosinophil recruitment [23]. High levels of IFNγ are associated with exacerbations and increased asthma severity [24]. Thus, the inhibitory effect of MnTE-2-PyP on IFNγ, IL-4, and IL-5 production may be part of the mechanism by which this compound attenuates airway inflammation observed in a mouse model of allergic asthma [1].

Pre-treatment of either DC or Th2 cells with MnTE-2-PyP had no effect on the proliferation of Th2 cells. The cell proliferation was inhibited only when MnTE-2-PyP was present in the culture media during stimulation, regardless of stimulatory methods used (DC-stimulated or anti-CD3/CD28 antibodies stimulated Th2 cells), and regardless of OVA_323–339_ peptide concentrations. These results imply that ROS release is necessary for stimulation and proliferation of Th2 cells and that when ROS is scavenged by MnTE-2-PyP the Th2 cells do not respond to stimulation. This essential role of ROS in CD4^+^ T-cell proliferation has been demonstrated in chronic beryllium disease in which a catalytic antioxidant MnTBAP reduces T-cell proliferation stimulated with beryllium [25].

The Th2 cells from the culture exposed to MnTE-2-PyP do not appear to be tolerogenic since removal of MnTE-2-PyP from the culture reestablishes Th2 cell proliferation (Figure 2). Thus, treatment with MnTE-2-PyP may not have long term global suppression of T cells but may be critical for short term treatment rendering antigen-specific T cells hyporesponsive. The exact mechanism of how MnTE-2-PyP exerts the anti-proliferative effect on Th2 cell is unknown. However, one possibility is the suppression of CD25 expression on T cells (Figure 3), indicating a decrease in T cell activation. These data suggest that administration of MnTE-2-PyP may be useful in suppressing T cell-mediated immune responses because MnTE-2-PyP suppresses the response to different innocuous antigens displayed by DC. Another possible explanation is the suppression of co-stimulatory molecules on DC (Figure 4), which may interfere with DC-T cell interactions. However, it is unclear how MnTE-2-PyP interferes directly with DC-Th2 cell interactions. We hypothesize that ROS scavenging reduces the presence of cell surface markers, CD40 in particular, which is supported by the results in Figure 4. Future experiments will investigate the effect of MnTE-2-PyP on the interactions between DC and Th2 cell surface molecules such as CD40-CD40L and B7-CD28 interactions.

The results from this study are similar to the findings by Tse *et al*. using a diabetic mouse model. They showed that MnTE-2-PyP inhibits CD4^+^, APC, and CD8^+^ activation and proliferation [16,18,26,27]. They also demonstrated a reduction in pro-inflammatory cytokine production in the presence of MnTE-2-PyP [26,27]. A recent study has shown that another antioxidant enzyme, glutathione peroxidase, suppresses Th2 and Th17 cell development in an allergen-induced asthma model [28]. In addition, elevated mitochondrial SOD disrupts normal T cell development and impairs adaptive immune responses in an influenza infection [29]. Therefore, there is mounting evidence that the oxidative environment surrounding the adaptive immune system alters the differentiation and proliferation of Th2 cell types in a variety of inflammatory diseases.

DC treated with MnTE-2-PyP, have increased basal IL-12 production. The level of IL-12 was markedly enhanced in OVA-activated DC treated with MnTE-2-PyP as compared to OVA-activated DC alone. These results indicate that MnTE-2-PyP activate the DC, since IL-12 is a marker of activated DC [22]. Because MnTE-2-PyP enhances IL-12 production by OVA-stimulated DC, this could be a reason for the decrease in production of IL-4 and IL-5 (Figure 1). In addition, MnTE-2-PyP also down-regulates co-stimulatory molecules (Figure 4), which may explain the immunosuppressive effect (cytokine production and cell proliferation) of MnTE-2-PyP. The fact that only MnTE-2-PyP treated DC but not MnTE-2-PyP treated Th2 cells (Figure 1, closed bars) inhibit cytokine production, suggests that MnTE-2-PyP acts directly on the DC to alter T cell activity. Piganelli *et al.* found that IL-12 was unchanged in OVA treated DO11.10 cells in the presence of MnTE-2-PyP [27]. The difference in IL-12 expression between these two studies may be due to difference in concentration of OVA used in the two experiments. The effect of MnTE-2-PyP on IL-12 production by DC appears to be regulated by ROS. Recent work published by Jendrysik *et al.* [30] demonstrates that ROS deficient DC produce more IL-12 than the wild-type. A reduction in ROS levels leads to hyperphosphorylation of p38, a transcription factor, which enhances IL-12p70 expression. MnTE-2-PyP treated DC mimics ROS-deficient conditions by scavenging ROS. We believe the high expression of IL-12 in MnTE-2-PyP is due to increased P-p38 thereby increasing IL-12p70 production. The enhancement of IL-12 production may partly explain the decreased in IL-4 and IL-5 productions by OVA-specific Th2 cells (Figure 1).

## 3. Experimental Section

### 3.1. Generation of Dendritic Cells from Bone Marrow Progenitors

DC were generated from bone marrow as described previously [20,21]. Briefly, at day 0, bone marrow cells were harvested from femurs and tibiae of BALB/c mice, resuspended in culture medium containing 500 units/mL of GM-CSF and 500 units/mL of IL-4 (BD Pharmingin, San Diego, CA). Cells were seeded at 2.5 × 10^5^ cells/mL in six-well plates. On day 3, an additional GM-CSF (250 units/mL) was added to the plates. On day 6, half of the culture supernatant was collected and centrifuged. The cell pellet was suspended in culture medium containing GM-CSF (250 units/mL), and reseeded into the original plates. On day 7, cell cultures were harvested, sorted for CD11c^+^ cells using magnetic beads directly conjugated with antibody to CD11c (Miltenyi Biotec, Auburn, CA), and resuspended in culture medium. DC were divided into two groups and cultured either in the presence or absence of 30 μM MnTE-2-PyP (DC_t_ and DC_ut_, respectively) for an additional 3 days.

### 3.2. Generation of OVA-Specific Th2 Cells

T lymphocytes were enriched from the spleens of DO11.10 mice using a CD4^+^ enrichment kit (Miltenyi Biotec, Auburn, CA), according to the manufacturer’s instructions. CD4^+^ cells were cultured with DC in a ratio of 20:1 in the presence of 1.5 μM OVA peptide, anti-IL-12 (10 μg/mL) and IL-4 (10 ng/mL) for 5 days in 96-well plates. Cells were then collected and transferred to a 12-well plate pre-coated with anti-CD3 (0.5 μg/mL) and anti-CD28 (0.5 μg/mL) antibodies and continued culturing with 1.5 μM OVA peptide, in the presence of anti-IL-12 and IL-4 for an additional 7 days. The OVA-specific Th2 cells were divided into two groups for culturing either with or without 30 μM MnTE-2-PyP for an additional 3 days.

### 3.3. DC-Dependent T Cell Production of Cytokines

MnTE-2-PyP-untreated Th2 (Th2_ut_) or treated Th2 cells (Th2_t_) (2 × 10^5^ cells/well) were co-cultured with MnTE-2-PyP-untreated- or treated-DC (DC_ut_ and DC_t_, respectively) (1 × 10^4^ cells/well) in four different combinations (Th2_ut_-DC_ut_; Th2_t_-DC_ut_, Th2_ut_-DC_t_ and Th2_t_-DC_t_) in 96-well plates in the presence of OVA_323–339_ peptide (3 μM) for 96 h. Levels of IL-4, IL-5, and IFNγ in supernatants from the cultures were determined using OptEIA kits (BD Pharmingen), according to the manufacturer’s instructions.

### 3.4. Antigen-Specific Th2 Cell Proliferation

To assess the effect of MnTE-2-PyP on antigen specific Th2 cell proliferation, MnTE-2-PyP-treated or untreated Th2 cells were co-cultured with DC (treated or untreated) in four different combinations as described above. In separate experiments, treated- and untreated-Th2 cells were cultured in pre-coated 96-well plate with anti-CD3 (0.5 μg/mL) and anti-CD28 (0.5 μg/mL) antibodies with titrated doses of OVA peptide without DC. Th2 cells were cultured either with or without MnTE-2-PyP (30 μM) for 96 h. [^3^H]thymidine (1 μCi/well) was added during the last 18 h of culturing. Proliferation was measured in a β-scintilation counter (Packard TopCount NXT, Packard Instrument, Meriden, CT).

### 3.5. FACS Analysis of Cell Surface Markers

To analyze cell surface molecules expression by flow cytometry, CD11c^+^ DC were stimulated with 100 μg/mL endotoxin-depleted OVA in the presence or absence of MnTE-2-PyP (30 μM) or kept in media alone as a control for 42 h. Supernatants were collected and stored at −20 °C for IL-10 and IL-12 assays. Cells were blocked with anti-Fc receptor, and incubated with conjugated fluorochrome antibodies specific for MHC class II/I-A^b^ (M5/114.2), CD40 (3/23), CD54/ICAM-1 (3E2) CD80 (16-10 A1) and CD86 (PO3) for 15 min at room temperature. Cells were washed twice in FACS buffer containing 2% FCS in PBS. Twenty thousand events of live cells were analyzed for each sample; propidium iodide positive cells were excluded.

To analyze IL-2 receptor-chain (CD25) expressions on Th2 cells, Th2 cells were cultured in the presence or absence of 30 μM MnTE-2-PyP (Th2_t_ or Th2_ut_, respectively) for 3 days. Cells were then transferred to a pre-coated 96-well plate with anti-CD3 and anti-CD28 antibodies and 1.5 μM of OVA_323–339_ peptide in the presence or absence of MnTE-2-PyP for an additional 96 h. All four combinations of Th2 cells were stained for 15 min at room temperature with antibodies specific for CD25 (7D4) and CD4 (GK1.5) specific antibodies as described above. Twenty thousand events of live cells were analyzed for each sample.

### 3.6. IL-10 and IL-12 Cytokine Assays

Levels of IL-10 and IL-12 (p70) in the supernatant from DC cultures were determined using OptEIA kits (BD Pharmingen), following manufacturer’s instructions.

### 3.7. Statistical Analysis

Results from all groups were compared by ANOVA followed by Tukey-Kramer post hoc analysis. A *p* value less than 0.05 was considered statistically significant. Results for all measurements are expressed as mean ± SEM.

## 4. Conclusions

In summary, it is likely that the SOD mimetic, MnTE-2-PyP, alters Th2 cell immune responses by down regulating co-stimulatory molecules on DC and by inhibiting the up-regulation of CD25 on Th2 cells in an allergic model. It is also possible that MnTE-2-PyP has a direct influence on DC-Th2 cell interactions. Thus, it would be interesting to investigate whether immunological synapses between DC and Th2 cells are altered by MnTE-2-PyP. These findings corroborate other findings showing that redox environment controls the adaptive immune system. Since there are many diseases associated with uncontrolled pro-inflammatory responses, antioxidants may prove to be beneficial in dampening the adaptive immune response. This study shows that MnTE-2-PyP is able to down regulate the Th2 immune response in an allergic asthma model by inhibiting DC and Th2 cell interactions.
